# Crack-Mitigating Strategy in Directed Energy Deposition of Refractory Complex Concentrated CrNbTiZr Alloy

**DOI:** 10.3390/ma18153653

**Published:** 2025-08-04

**Authors:** Jan Kout, Tomáš Krajňák, Pavel Salvetr, Pavel Podaný, Michal Brázda, Dalibor Preisler, Miloš Janeček, Petr Harcuba, Josef Stráský, Jan Džugan

**Affiliations:** 1 COMTES FHT, Průmyslová 996, 334 41 Dobřany, Czech Republic; pavel.salvetr@comtesfht.cz (P.S.); pavel.podany@comtesfht.cz (P.P.); michal.brazda@comtesfht.cz (M.B.); jan.dzugan@comtesfht.cz (J.D.); 2 Department of Metals and Corrosion Engineering, University of Chemistry and Technology, Technická 6, 166 28 Praha 6, Czech Republic; 3 Faculty of Mathematics and Physics, Charles University, Ke Karlovu 3, 121 16 Praha 2, Czech Republic; tomas.krajnak@matfyz.cuni.cz (T.K.); dalibor.preisler@matfyz.cuni.cz (D.P.); milos.janecek@matfyz.cuni.cz (M.J.); petr.harcuba@matfyz.cuni.cz (P.H.); josef.strasky@matfyz.cuni.cz (J.S.); 4 Research Centre, University of Žilina, Univerzitná 1, 01026 Žilina, Slovakia

**Keywords:** refractory metals, refractory complex concentrated alloys, high-entropy alloys, additive manufacturing, directed energy deposition

## Abstract

The conventional manufacturing of refractory complex concentrated alloys (RCCAs) for high-temperature applications is complicated, particularly when material costs and high melting points of the materials processed are considered. Additive manufacturing (AM) could provide an effective alternative. However, the extreme temperatures involved represent significant challenges for manufacturing defect-free alloys using this approach. To address this issue, we investigated the preparation of a CrNbTiZr quaternary complex concentrated alloy from an equimolar blend of elemental powders using commercially available powder-blown L-DED technology. Initially, the alloys exhibited some defects owing to the internal stress caused by the temperature gradients. This was subsequently resolved by optimizing the deposition strategy. SEM, XRD and EDS were used to analyze the alloy in the as-deposited condition, revealing a BCC phase and a secondary Laves phase. Furthermore, Vickers hardness testing demonstrated a correlation between the hardness and the volume fraction of the Laves phase. Finally, successfully performed compression tests confirmed that the prepared material exhibits high-temperature strength and therefore is promising for high-temperature application under extreme conditions.

## 1. Introduction

Refractory complex concentrated alloys (RCCAs) containing multiple principal elements have been proposed as a new group of materials capable of withstanding extreme mechanical loads and temperatures involved in modern engineering applications such as gas turbines, nuclear reactors or even space vehicle re-entry [1]. However, the development of RCCAs into viable components and devices faces several formidable challenges [2]. Achieving homogeneous microstructures is challenging because of the disparate melting points and atomic sizes of the constituent elements [3]. These challenges are exacerbated by the limited availability and high cost of refractory metals, as well as the scarcity of suitable manufacturing equipment capable of consistently and safely operating at elevated temperatures. Hence, only a few of such alloys have been successfully produced and evaluated [4].

One potential solution for the difficulties associated with RCCA development is to employ high-throughput fabrication techniques, such as 3D printing methods, in which localized melting of the material is achieved using lasers or electron beams [5,6,7,8,9,10,11,12,13]. Laser-directed energy deposition (L-DED), a workhorse of additive manufacturing (AM), enables the rapid synthesis and evaluation of a wide range of compositional variants of complex alloy systems [7,11], potentially accelerating the material development process [10]. Because of the layer-by-layer deposition process, the chemical composition of each printed layer can be precisely controlled, thereby facilitating the fabrication of compositional gradients within a single part [14]. Moorehead et al. stated that AM could dramatically accelerate material development, potentially reducing the processing time by up to 100 times compared with traditional manufacturing methods [15].

AM of RCCAs can be achieved using either pre-alloyed powders or a blend of elemental powders. The latter option is particularly appealing because of its cost-effectiveness and powder quality. However, the use of elemental powders in DED requires more intensive optimization of the deposition parameters to produce a defect-free homogenous material [16]. The first system prepared by DED from elemental powders was the now-renowned MoNbTaW alloy, which was fabricated both as a thin-wall structure [17] and as a bulk sample [7,13].

Nevertheless, the possible applications of RCCAs require a focus on their superior high-temperature oxidation and corrosion resistance, which includes the use of elements capable of forming stable oxide coatings, such as Al, Cr, Ti and Si [18,19,20]. However, compared with refractory metals, these elements have significant disparities between their melting points and atomic radii, resulting in the formation of brittle intermetallic phases that complicate the deposition and deteriorate mechanical properties [21,22,23,24]. Furthermore, the high thermal gradients experienced during cooling can lead to residual stresses and defects in the final product, including cracks, residual porosity, lack of fusion and significant elemental segregation [25]. Dobbelstein et al. synthesized Ti-containing columnar samples of HfNbTaTiZr [9,11] using DED, as well as a graded sample of the quaternary subsystem NbTaTiZr [10] in the form of a thin wall. However, these studies reported significant residual porosity, which was attributed to the high oxygen affinity of the input powders. To the best of our knowledge, no study has reported the DED of bulk defect-free RCCAs containing reactive elements.

In this study, we developed a methodology for the L-DED of CrNbTiZr alloy using a mixture of elemental powders. By optimizing the processing parameters and managing the heat conduction to the platform, we successfully fabricated crack-free bulk samples. In addition, we conducted a microstructural characterization and evaluated the mechanical properties under compression at both ambient and elevated temperatures.

## 2. Materials and Methods

### 2.1. Powder Mixture Characteristics and Preparation

**Figure 1:**
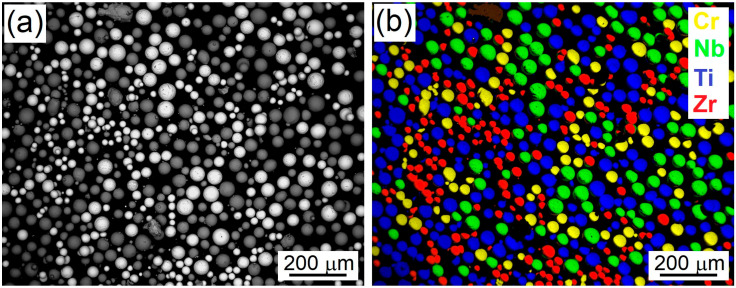
(**a**) Backscatter SEM image and (**b**) EDS map of the blend of elemental powders used for the sample deposition. The figure is sourced from [26].

Equimolar elemental powder mixture of spherical Cr, Nb, Ti and Zr was deposited using commercially available powder-blown L-DED apparatus Insstek MX-Lab (Insstek, Daejeon, South Korea) equipped with an Ytterbium-doped fiber laser generating a wavelength of 1070 nm and 400 μm beam diameter with Gaussian profile. Chemical compositions of individual powders, particle size distribution and their manufacturers are listed in Table 1. A 500 g blend of the powders in equimolar ratios was prepared by intensive mixing in a 3D shaker–mixer Turbula (WAB, Muttenz, Switzerland) for one hour. The blend was completely re-mixed three times. The SEM-BSE image of the powder blend and the EDS map are shown in Figure 1. A powder feeding rate of 0.5 g/min was used for all deposition strategies. The powder feeding is controlled by the vibration system, which theoretically enables a linear variation in the feeding rate in the range of 0.05 g/min to 20 g/min. Nevertheless, the feeding rate was optimized to prevent a high amount of back-reflection to the laser system, which interrupted the deposition. A precise laboratory balance Kern PBF 200-3 (KERN & SOHN, Balingen-Frommern, Germany) was used to calibrate the system settings. The powder feeding was also enhanced by argon gas flow whose flow rate was set to 3 L/min.

The deposition chamber of the PB L-DED apparatus was filled with a protective argon atmosphere of 99.9999% purity. During the deposition process, the chamber was continuously purged to maintain an oxygen concentration superior to 100 ppm. This concentration was monitored using an electrochemical-based built-in sensor, which allowed the machine to automatically control the gas flow based on the sensor readings. Additionally, the melt pool received further protection from the argon gas flow through the nozzle module, coaxially placed with the laser beam to surround the fed powder. The flow rate was maintained at 8 L/min.

### 2.2. Directed Energy Deposition of the Experimental Material

**Figure 2:**
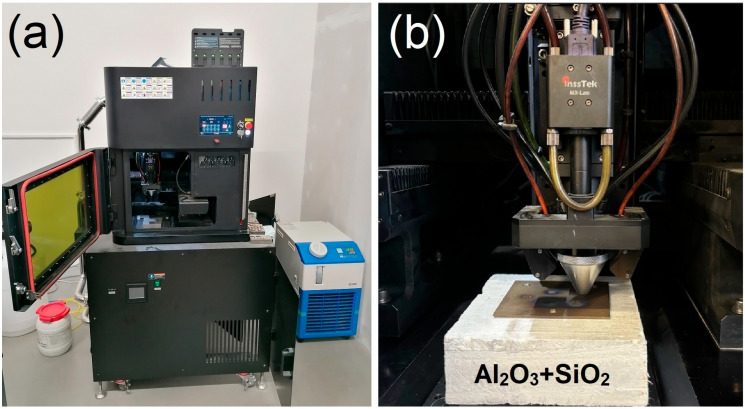
(**a**) Insstek MX-Lab deposition system, (**b**) experimental setup used for deposition on thermally insulated platform.

**Figure 3:**
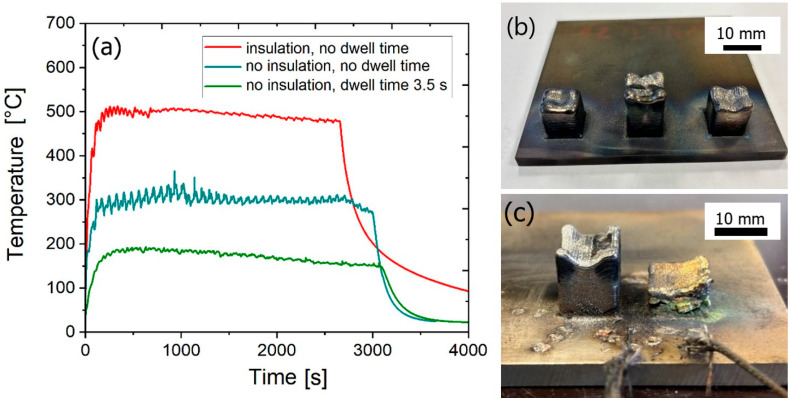
(**a**) Temperature evolution during the sample deposition at a laser power of 500 W with and without an insulated platform, (**b**) samples deposited via the optimized crack-mitigating strategy (insulated platform, no dwell time), and (**c**) samples deposited on a conductive platform.

To optimize the deposition conditions, the temperature evolution of the platform (Ti Grade 2, 95 × 95 × 5 mm) was measured using thermocouples welded next to the deposited samples. Placing the titanium platform on a 40-mm-thick ceramic fiber insulating platform Ultraboard 1850-400 (80% Al_2_O_3_ + 20% SiO_2_) (Schupp, Aachen, Germany) with a thermal conductivity of 0.34 W∙m^−1^∙K^−1^ was found to effectively suppress heat transfer to the permanently cooled working table of the machine which allows for minimizing cooling rates. Additionally, to achieve greater heat accumulation in the sample, the dwell time between the deposition of adjacent layers was turned off. This deposition strategy proved to reduce negative effects of rapid cooling, such as excessive internal stress, which are known to result in cracking of brittle materials such as RHEAs [27]. The Insstek MX-Lab apparatus and experimental setup used for deposition on a thermally insulated platform are shown in Figure 2. The temperature evolution during the deposition of bulk samples with and without an insulated plate having nominal dimensions of 12 × 12 × 12 mm is shown in Figure 3a. Images of the samples deposited on insulated and conductive platforms are shown in Figure 3b and Figure 3c, respectively.

The laser power of 500 W was used, providing the maximum possible output from the built-in laser system of the apparatus. The laser scanning velocity was set to 850 mm/min in all depositions. Other studies [5,9,10,16,17] proved that reducing the scanning velocity is beneficial for improving the lack-of-fusion defects and chemical homogeneity. However, experiments with reduced scanning velocity introduced several technical complications; in particular, the increased number of reflections back to the optical module, which led to interruptions of the deposition process resulting in cracking of samples, and ultimately damaging the DED device. The laser beam diameter was 400 μm, and the laser scanning trajectory followed a ZigZag link CF/CFC pattern with a 90° rotation between adjacent layers. The term “link” indicates that the ZigZag scans are continuously connected, and the laser is not switched off at any point during the deposition, thereby minimizing the dwell time. The designation “CF/CFC” means that for odd-numbered layers, the contour of the square-shaped layer was deposited first, followed by filling using ZigZag scans, whereas for even-numbered layers an additional contour was deposited. The hatch distance, which is the distance between adjacent laser scans of the filling, was set to 300 μm, resulting in a 100 μm overlap. The nominal layer height, which is the shift in the *Z*-axis after the completion of each layer, was set to 150 μm. The deposition parameters are summarized in Table 2.

### 2.3. Material Characterization

**Figure 4:**
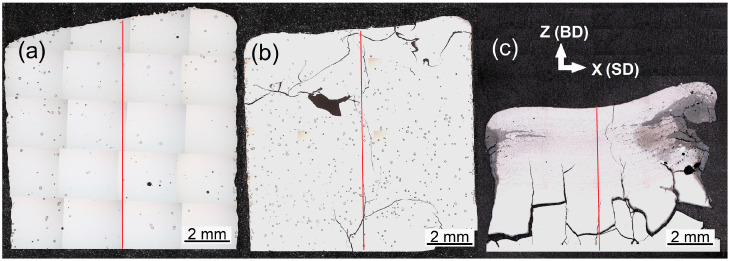
Overview (low magnification) light micrographs of the samples deposited by a constant laser power of 500 W (**a**) on a thermally insulated platform, no dwell time, (**b**) on a conductive platform, no dwell time, and (**c**) on a conductive platform, dwell time of 3.5 s.

The deposited material was extracted from the platform and sectioned in the XZ plane, which corresponds to the building direction, using an electrical discharge machine. The surface intended for microstructural analysis was prepared using standard metallographic methods, which included grinding with SiC papers followed by polishing. The final polishing step involved the use of a 20% hydrogen peroxide solution in an OP-S colloidal silica suspension for 7 min. An overview of the impurities and defects was conducted using an optical microscope (Zeiss Axio Observer Z1m, Oberkochen, Germany) at 50× magnification. For subsequent microstructural observations and basic analysis of chemical composition, JEOL 6380 (Jeol, Tokyo, Japan), TESCAN VEGA 3 LMU, FEI Quanta 200 and Apreo 2 (Thermo Fisher Scientific, Waltham, MA, USA) scanning electron microscopes (SEM) equipped with EDAX and Oxford Instruments (Abingdon, UK) EDS detectors were used. The EDS stripe profile was evaluated from the discrete rectangular EDS pattern consisting of perpendicular lines of the length of 266 µm determined with a step of 50 µm along the *Z*-axis (red line in Figure 4). Furthermore, EDS elemental mapping was conducted on the sample deposited using the optimized crack-mitigating strategy (insulated platform, no dwell time) of approximately 2.5 mm from both the bottom and top surfaces, which corresponded to the positions of the extracted specimens for the compression test.

The crystal structure was determined using a Brukker D8 Discover X-ray diffractometer (Billerica, MA, USA) with Cu Kα radiation, a 2 mm spot size, and a scattering range of 20–140°. XRD was performed in the bottom, middle and top sections of the sample. Scanned spots in the bottom and top sections corresponded to the extraction positions of samples used for the compression test and the determination of the chemical composition was complemented by hardness testing performed along three lines with 3 mm distance (along the *X*-axis) and 250 µm distance between individual indents (along the *Z*-axis). The line corresponding to x = 0 refers to the central part of the sample. The individual microhardness values were evaluated using an automatic Struers Durascan 50 (Struers, Ballerup, Denmark) according to the ISO 6507 standard for Vickers HV1 [28].

The mechanical properties at RT, 600 °C and 800 °C of the crack-free compact sample deposited with insulation and no dwell time (see Figure 3a and Figure 4a) were investigated under compression loading with the constant crosshead velocity corresponding to an initial strain rate of 10^−3^ s^−1^ using a Zwick Roell Z250 (Singapore) machine with a maximum load cell capacity of 250 kN. Three samples were tested at each temperature. The samples tested at elevated temperatures were held for 10 min at the temperature before the test. Three cylindrical samples (diameter: 4 mm; height: 6 mm) were prepared by electrical discharge machine from each section of the deposited cube. The axis of the specimen for compression test was parallel to the *Y*-axis. The strain measurement was performed using a contactless video extensometer, employing facet sizes of 50 × 100 pixels and an optical system from Zwick Roell.

## 3. Results

### 3.1. Microstructure Comparison of Samples Deposited on Conductive and Thermally Insulated Platform

The characteristic cross-sections of deposited specimens are shown in Figure 4. Samples deposited on the conductive platform contain numerous cracks, see Figure 4b,c. Despite being crack-free, the sample deposited via the optimized crack-mitigating strategy (insulated platform, no dwell time, Figure 4a) contained residual undissolved or only partially dissolved powder particles, as well as pores. Image analysis revealed that the pores occupied approximately 0.7% of the scanned area. Nevertheless, the results show that the number of undissolved Nb particles and the presence of other defects are inversely proportional to the temperatures reached during the deposition.

### 3.2. Chemical Composition of the Samples Along Z-Axis

**Figure 5:**
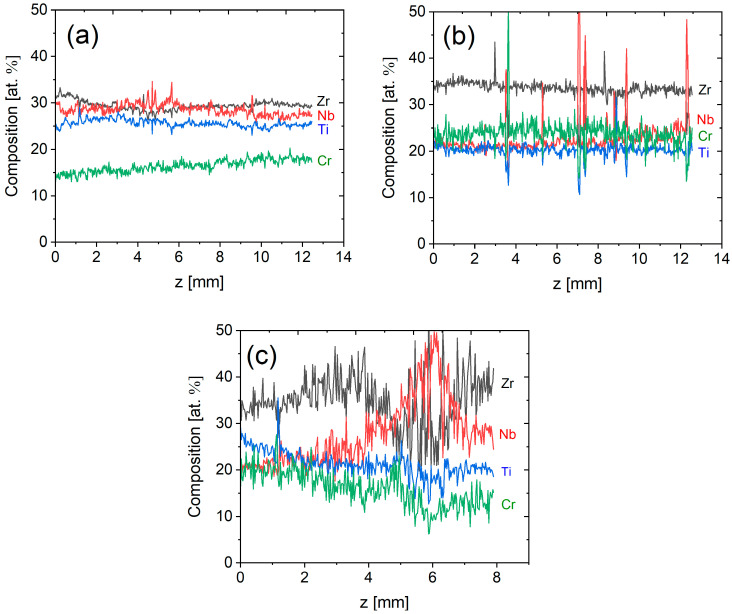
Chemical composition evolution along the Z-axis of the samples deposited by a constant laser power of 500 W (**a**) on an insulated platform, no dwell time, (**b**) on a conductive platform, no dwell time, and (**c**) on a conductive platform, dwell time 3.5 s, evaluated from the EDS strip.

Distinct deviations from the envisioned homogeneous equimolar composition were observed in the EDS strip measurements shown in Figure 5. The average chemical compositions in the top and bottom sections of the sample deposited via the optimized crack-mitigating strategy (insulated platform, no dwell time) as determined from EDS maps are listed in Table 3.

Figure 5a shows that Cr content in the sample deposited via optimized crack-mitigating strategy (insulated platform, no dwell time) is significantly lower than the initial content of the powder mixture (25 at. %). Cr concentration gradient from 15 at. % measured in the bottom section to 21 at. % in the top section is apparent and is probably caused by Cr evaporation as discussed below. The other elements exhibit a constant evolution along the *Z*-axis. The compositional evolution of the sample deposited without dwell time on the conductive platform is demonstrated in Figure 5b. In this sample, no Cr concentration gradient was observed. However, a slight increase in the Nb concentration was observed on the top part. In addition, distinct peaks were apparent in all concentration profiles, particularly in the Nb profile. These observed peaks indicate the presence of unmelted particles in the deposited track, as confirmed by optical microscopy. Despite the overall consistent compositional evolution, a deviation from the initial equimolar concentration in the Zr content was observed. For the sample deposited on the conductive platform and dwell time of 3.5 s (the profile presented in Figure 5c), severe disparities from the equimolar composition and inhomogeneities were observed.

These results indicate unambiguously that employing a thermally insulated platform and minimizing the dwell time significantly improves the chemical homogeneity of the resulting material. Nevertheless, a higher number of thermal cycles leads to the evaporation of elements with lower melting points, as discussed in more detail below.

### 3.3. Microstructural Dependence on Processing Conditions

**Figure 6:**
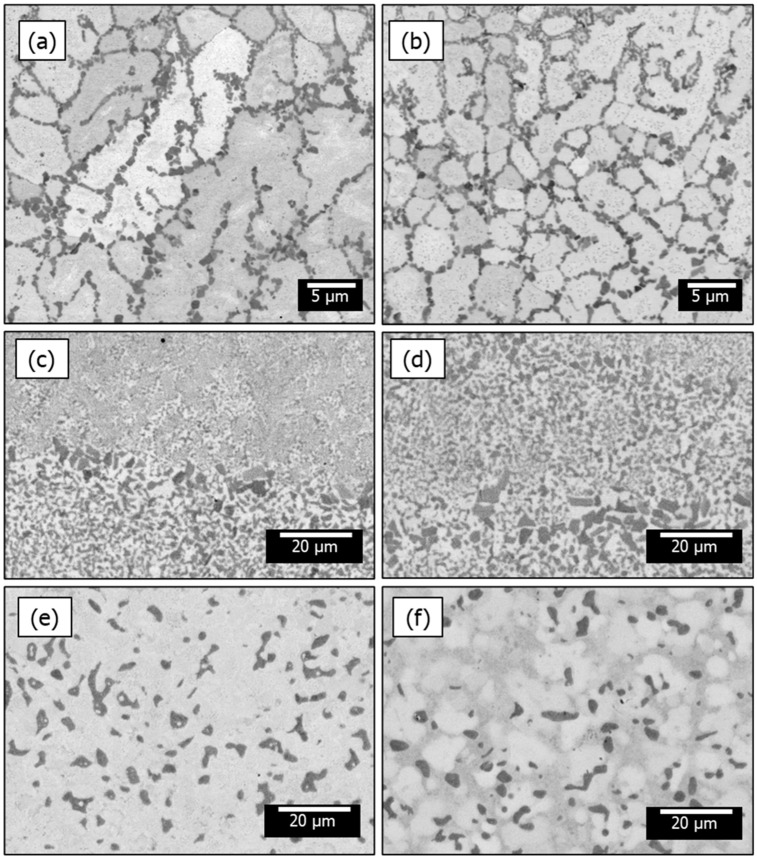
Backscatter SEM images of the (**a**,**b**) sample deposited via the optimized crack-mitigating strategy (insulated platform, no dwell time), (**c**,**d**) sample deposited on a conductive platform without the dwell time, and (**e**,**f**) sample deposited on a conductive platform, dwell time 3.5 s.

Figure 6 presents a comparison of the microstructural evolution across different processing conditions, in the middle part of deposited samples. Two images are shown for each sample. Two regions with distinct contrasts were evident in all samples. The sample deposited via the optimized crack-mitigating strategy (insulated platform, no dwell time) contained a dendritic structure, with bright dendritic regions and dark inter-dendritic regions comprising cubic Laves C15 phase as confirmed by results of other authors [3,18,23,24]. In contrast, the sample deposited on the conductive platform featured a bright matrix phase interspersed with randomly oriented dark-Laves-phase precipitates. These findings differ from the conventional microstructure of additively manufactured samples, which typically display visible melt pools. Table 4 provides details on the chemical compositions of such bright and dark regions in the bottom and top sections of the sample deposited via the optimized crack-mitigating strategy. The listed data were averaged from 30 individual EDS points scanned in each region.

### 3.4. Phase Composition

**Figure 7:**
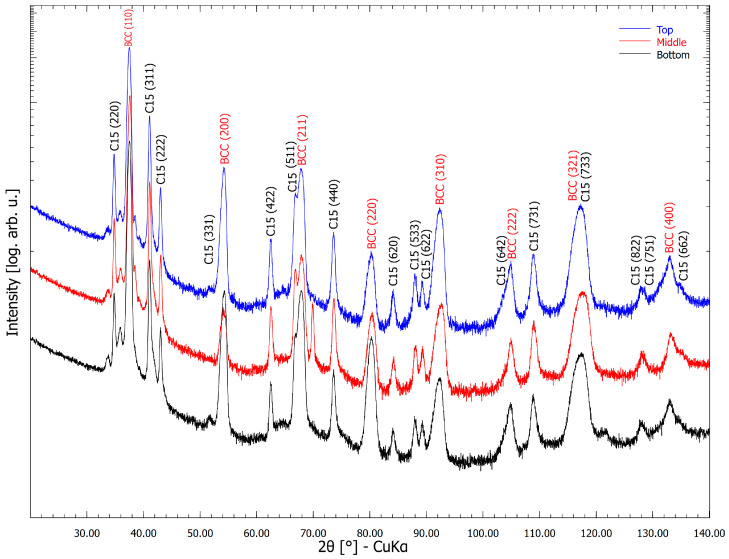
X-ray diffraction patterns along the build direction of the sample deposited on the thermally insulated platform without a dwell time. The intensity axis was plotted on a logarithmic scale for better clarity.

The X-ray diffraction patterns acquired in three regions along the Z-axis of the as-deposited sample are shown in Figure 7. The structures of all three studied regions consist of two phases: the BCC and cubic Laves (C15) phases. The lattice parameter of the BCC phase was determined to be 338.1 pm and that of the C15 Laves phase 727.5 pm. Note that the refined lattice parameter for the cubic Laves C15 phase with a stoichiometric composition Cr_2_Zr is 720.86 pm [29]. However, in this case, the Laves phase also contains dissolved Nb, Ti and Zr atoms. Based on the results of [3,18,23,24], it is confirmed that the BCC peaks belong to the bright dendritic regions in Figure 6, while the Laves C15 structures are located in the dark inter-dendritic regions. A minor increase of ~2.4% in Laves phase concentration was observed in the top section compared to the bottom one.

### 3.5. Mechanical Properties

**Figure 8:**
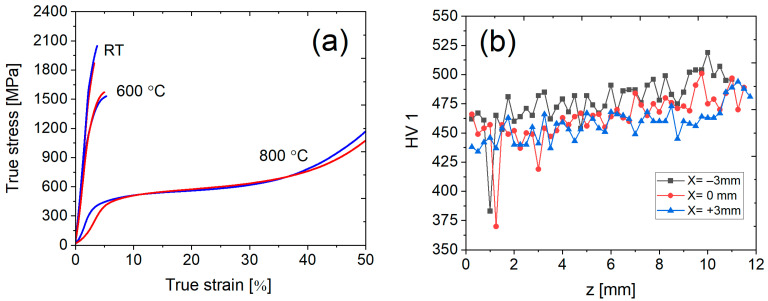
(**a**) Compression stress–strain curves at RT, 600 °C and 800 °C, and (**b**) microhardness evolution across the height (*Z*-axis) of the sample deposited via the optimized strategy (insulated platform, no dwell time).

Due to the observed different chemical compositions along the *Z*-axis, compressive specimens were extracted from the bottom and top sections to account for discrepancies in the mechanical properties. The compressive stress–strain curves of samples tested at RT, 600 °C and 800 °C are shown in Figure 8a. The evaluated compressive 0.2% offset yield stress, maximum compressive strength and strain to fracture are summarized in Table 5.

Significantly higher yield strengths were achieved in the specimens extracted from the top section of the sample. The specimens deformed at RT exhibited yield stress of 1669 MPa, compressive strength of 1810 MPa and strain to fracture of 3.4% for the bottom section, while for the top section, the determined values were the following: the yield stress 1800 MPa, the compressive strength 1973 MPa and total strain 3.8%. At 600 °C, both evaluated samples maintained a yield strength exceeding 1000 MPa, despite fracturing at approximately 5% strain. Both samples tested were ductile at 800 °C with strains exceeding 50%. The yield strengths were 388 MPa and 436 MPa for the bottom and top sections, respectively. Steady state plastic flow was observed for the specimens compressed at 800 °C.

The increasing trend of Vickers hardness profile shown in Figure 8b correlates well with the Cr concentration. This phenomenon is associated with different amounts and morphology of the hard Laves phase, as explained in the discussion section. There are notable deviations between the values of the three indentation lines, but the overall increasing trend is the same for all measured lines. The measured differences between the values in the bottom and top sections were approximately 50 HV1. The indents that demonstrated a low Vickers hardness value of ~375 HV1 correspond to the positions of undissolved Nb particles, as confirmed by OM.

## 4. Discussion

The present study demonstrates that a strategic combination of deposition-parameters optimization and thermal management enables the fabrication of a crack-free bulk CrNbTiZr RCCA via L-DED using elemental powders. This represents a significant advancement, as prior efforts involving DED of similar systems have typically been limited to thin-wall geometries and have often suffered from severe cracking, substantial residual porosity, or incomplete fusion [7,8,9,10,11,13,17]. Our results indicate that insulating the build platform and minimizing the dwell time between the layers elevates the overall temperature of the sample, effectively preventing the formation of macrocracks. It can be attributed to the fact that the deposition on insulated substrate reduces temperature gradients created at the edge of the melt pool which in turn decreases the strain concentrations and suppresses the formation of cracks. In contrast, samples deposited on the thermally conductive platform exhibited pronounced cracking. A laser-induced pre-heating temperature of approximately 500 °C was determined to be adequate to achieve this effect.

Despite using maximal attainable laser power output of 500 W and the mentioned thermally insulated platform, there is still a notable amount of undissolved Nb and Zr particles present in the microstructure. Nonetheless, their presence highlights a remaining challenge in achieving complete melting of elemental powders in L-DED [8]. Interestingly, while undissolved particles were still present, their detrimental effect on compressive yield strength appears to be low. The compression yield strength achieved in the studied material is comparable to that of the equimolar CrNbTiZr alloy that was subjected to hot isostatic pressing (HIP) at 1200 °C and 207 MPa for 1 h, followed by a 24 h annealing at 1200 °C [3,21,22,24]. This resemblance in properties is important, since achieving such mechanical properties directly from the additive manufacturing process, without requiring further heat treatments or HIP, offers considerable benefits in terms of cost and time savings.

The occurrence of undissolved particles might potentially be reduced by lowering the scanning velocity as reported in [16], or by incorporating additional remelting steps [17]. Decreasing the scanning velocity and incorporation of remelting steps has also been tested but led to technical difficulties with high back reflections as discussed in the methodology section. Further post-processing homogenization treatments may also help to reduce or eliminate such defects in future efforts.

Despite the success in achieving crack-free builds, some degree of deviation from equimolar composition was observed. Notably, a Cr concentration gradient was evident in the build direction, with lower Cr content at the bottom section and higher concentrations near the top. This phenomenon likely arises from different vaporization of Cr during prolonged laser exposure, particularly at the bottom layers that undergo more thermal cycling than the top ones, as shown in [30]. Such evaporation-related elemental redistribution has also been reported in other reactive RCCA systems [9,10,11]. Powder blend de-mixing during deposition can also be considered as a source of the observed inhomogeneities. This phenomenon can occur due to various factors, including differences in particle size, shape, density, and surface properties among the constituent powders. Nevertheless, further investigation of the specific powder characteristics is necessary to determine the likelihood and the extent of de-mixing in this case. Despite powder de-mixing presenting a viable concern in additive manufacturing using elemental powders, it has not been thoroughly explained in the literature on AM of RCCAs [7,9,10,15].

## 5. Conclusions

The successful fabrication of a crack-free RCCA CrNbTiZr through directed energy deposition using elemental powders represents a significant advancement in the additive manufacturing of high-performance materials. Even though undissolved Nb particles are present in the as-deposited condition, the achieved yield strength is comparable to that of the cast equimolar CrNbTiZr alloy subjected to hot isostatic pressing and a long-term annealing process [24]. This achievement highlights the potential of DED to produce high-performance materials in as-deposited condition having comparable or superior properties to those manufactured via conventional methods and subsequent thermomechanical processing.

## Figures and Tables

**Table 1:** Particle size distribution and measured impurities of the powders used for 3D printing.

Powder	Cr	Nb	Ti	Zr
Supplier	Stanford Advanced Materials, Lake Forest, CA, USA	Camex, Měšice, Czechia	AP&C, Boisbriand, QC, Canada	Camex, Měšice, Czechia
Particle size	50–150 µm	50–150 µm	45–150 µm	50–150 µm
Declared Purity	99.9%	99.9%	ASTM Grade 2	99.9%
Oxygen concentration (wt. %)	0.02	0.15	0.17	0.22
Nitrogen concentration (wt. %)	0.01	0.04	0.02	0.03

**Table 2:** Deposition parameters used in this study.

Laser Spot Diameter (µm)	Laser Power (W)	Scanning Velocity (mm/min)	Hatch Spacing (µm)	Layer Thickness (µm)	Powder Feed Rate (g/min)
400	500	850	300	150	0.5

**Table 3:** The average chemical composition of the top and bottom sections of the sample deposited via the optimized crack-mitigating strategy (insulated platform, no dwell time) determined by EDS mapping. The concentration values listed in the table are given in atomic %.

Section	Cr	Nb	Ti	Zr
Top	21 ± 2	28 ± 2	27 ± 3	23 ± 2
Bottom	17 ± 3	29 ± 2	28 ± 3	25 ± 2

**Table 4:** Average chemical compositions of the alloy constituents in the bottom and top sections of the sample deposited via the optimized crack-mitigating strategy. The concentration values in the table are given in atomic %.

Position	Bottom				Top			
	Cr	Nb	Ti	Zr	Cr	Nb	Ti	Zr
Dendritic regions (bright)	9 ± 1	38 ± 2	27 ± 1	26 ± 2	10 ± 1	34 ± 2	29 ± 1	27 ± 1
Inter-dendritic regions (dark)	29 ± 4	19 ± 3	22 ± 2	30 ± 2	33 ± 3	19 ± 2	20 ± 2	28 ± 1

**Table 5:** Offset yield stress σ_0.2_, maximum strength σ_max_ and fracture strain ε_f_ of the studied alloy at different temperatures and sections of the sample.

Temperature	Section	σ_0.2_ (MPa)	σ_max_ (MPa)	ε_f_ (%)
800 °C	Top	436 ± 21	1171 ± 58	>50%
	Bottom	388 ± 19	1077 ± 53	>50%
600 °C	Top	1237 ± 61	1496 ± 74	5.3 ± 0.3
	Bottom	1026 ± 51	1452 ± 72	5.6 ± 0.3
RT	Top	1800 ± 90	1973 ± 98	3.8 ± 0.2
	Bottom	1669 ± 83	1810 ± 90	3.4 ± 0.2

## Data Availability

The data that support the findings of this study are openly available in the Zenodo repository at htps://doi.org/10.5281/zenodo.16343902 under the CC-BY 4.0 license.

## Abbreviations

The following abbreviations are used in this manuscript:

HEA	High Entropy Alloy
DED	Directed Energy Deposition
L-DED	Laser Directed Energy Deposition
SEM	Scanning Electron Microscope
OM	Optical Microscope
EDS	Energy Dispersive Spectroscopy
CCA	Complex Concentrated Alloy
RCCA	Refractory Complex Concentrated Alloy
SD	Scanning direction
BD	Build direction
